# Deep short-read sequencing of chromosome 17 from the mouse strains A/J and CAST/Ei identifies significant germline variation and candidate genes that regulate liver triglyceride levels

**DOI:** 10.1186/gb-2009-10-10-r112

**Published:** 2009-10-13

**Authors:** Ian Sudbery, Jim Stalker, Jared T Simpson, Thomas Keane, Alistair G Rust, Matthew E Hurles, Klaudia Walter, Dee Lynch, Lydia Teboul, Steve D Brown, Heng Li, Zemin Ning, Joseph H Nadeau, Colleen M Croniger, Richard Durbin, David J Adams

**Affiliations:** 1 The Wellcome Trust Sanger Institute, Wellcome Trust Genome Campus, Hinxton, CB10 1HH, UK; 2 Mammalian Genetics Unit, MRC-Harwell, Harwell Science and Innovation Campus, Oxfordshire, OX11 ORD, UK; 3 Department of Genetics, Case Western Reserve University, Adelbert Rd, Cleveland, OH 44106-4955. USA

## Abstract

Genome sequences are essential tools for comparative and mutational analyses. Here we present the short read sequence of mouse chromosome 17 from the *Mus musculus domesticus *derived strain A/J, and the *Mus musculus castaneus *derived strain CAST/Ei. We describe approaches for the accurate identification of nucleotide and structural variation in the genomes of vertebrate experimental organisms, and show how these techniques can be applied to help prioritize candidate genes within quantitative trait loci.

## Abstract 2

Methods for accurate identification of nucleotide and structural variation using *de novo* short read sequencing of mouse chromosomes are described.

## Background

Mouse genetics has its origins with mouse fanciers who bred and maintained individuals lines of mice because of their unusual coat color or behavior [1,2]. The derivation of the classical laboratory mouse strains, however, only commenced early last century, driven by a desire to model human disease mechanisms [3]. It is now clear that these classical mouse strains are derived from a common pool of founders because haplotypes are shared, but have been shuffled, between strains prior to being fixed by inbreeding to select for desirable trait characteristics [4-7]. In contrast, wild-derived strains are highly divergent from these classical strains of mice, having been founded by inbreeding of wild-derived isolates [8].

In addition to the sequence of the reference strain, C57BL/6J, there are two large resources for genomic sequence of inbred mouse strains. Four laboratory strains were included by Celera in a whole genomic shotgun sequence of the mouse: A/J, DBA/2J, 129X1/SvJ, and 129S1/SvImJ [9]. The data consist of 27.4 million capillary sequence reads giving a total of 5.3× coverage of the mouse genome. Sequences are from both ends of size-selected 2-, 10-, and 50-kbp clones derived from randomly sheared mouse genomic DNA. Second, the National Institute of Environmental Health Sciences contracted Perlegen Sciences to resequence by hybridization 15 inbred mouse strains [10]. This set includes 11 classical strains (129S1/SvImJ, A/J, AKR/J, BALB/cBy, C3H/HeJ, DBA/2J, FVB/NJ, NOD/LtJ, BTBR T+tf/J, KK/HlJ and NZW/LacJ) and four strains derived from the wild (WSB/EiJ, PWD/PhJ, CAST/EiJ and MOLF/EiJ), which represent the *Mus musculus domesticus*, *Mus musculus musculus*, *Mus musculus castaneus *and *Mus musculus molossinus *subspecies. Unlike the Celera resource, the hybridization approach used by Perlegen does not generate sequence reads and can only reliably detect single nucleotide polymorphisms (SNPs). Furthermore, the hybridization technology queried only 1.49 billion bases of the reference genome. This represents about 58% of the C57BL/6J sequence that is non-repetitive. The Perlegen approach was also found to have a false negative rate as high as 50% [8]. Therefore, currently available sequence data lack the coverage and breadth of strains to make a universal resource.

Here we present the sequence and analysis of mouse chromosome 17 from A/J and CAST/Ei generated using massively parallel sequencing and illustrate the power of this approach for the accurate and sensitive analysis of mouse genome variation. We selected mouse chromosome 17 for this sequencing project because at 95 Mb in length it is a shorter mouse autosome, and has a distinct cytogenetic profile that allows it to be easily and cleanly flow sorted. Biologically, mouse chromosome 17 is of great interest because the mouse major histocompatibility complex (MHC) resides on this chromosome [11], as well as the murine *t*-complex, which in some wild-derived strains is responsible for transmission ratio distortion [12-14]. The strains A/J and CAST/Ei were selected for this pilot sequencing experiment because Celera has previously generated a significant number of shotgun capillary reads for A/J [9], and while A/J is related to C57BL/6J, and these strains share several haplotype blocks on chromosome 17 [8], CAST/Ei is highly divergent from the reference [8]. Indeed, the *M. m. castaneus *subspecific lineage is thought to have contributed less than 2% of the genomic material that makes up the classical laboratory strains of mice [8]. In addition, a large panel of congenic strains was derived from a consomic strain that has chromosome 17 from A/J substituted onto a C57BL/6J background [15]. These congenic strains have been extensively phenotyped to identify quantitative trait loci (QTLs) between C57BL/6J and A/J on chromosome 17. In particular, we were interested in using the A/J sequence of mouse chromosome 17 to refine a QTL reported to protect against high liver triglyceride levels [16]. We flow sorted mouse chromosome 17 from A/J and CAST/Ei and generated 22× and 34× coverage of these chromosomes, respectively. Using these data we identify and validate novel SNPs and structural variants between A/J, CAST/Ei, and the reference, and attempt a *de novo *assembly of these chromosomes. We illustrate that short read sequencing technology can be used to generate accurate assemblies of vertebrate experimental organisms such as mouse, allowing for the generation of a rich catalogue of variation between strains and the analysis of candidate genes within QTL intervals.

## Results

### Sequencing of mouse chromosome 17 using massively parallel short-read sequencing technology and mapping to the reference genome

**Figure 1:**
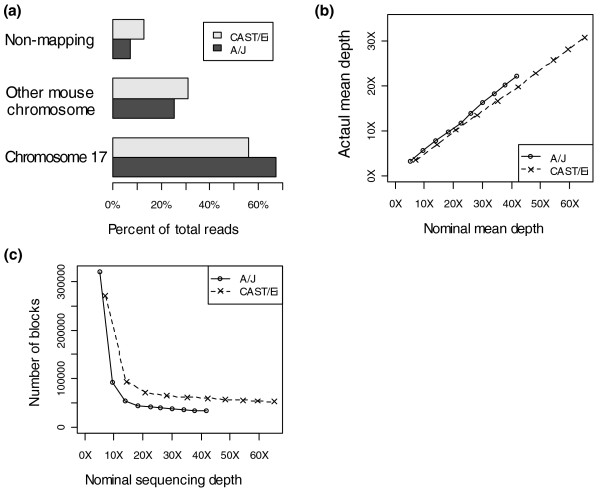
Mapping of short read sequence to the mouse genome. The MAQ algorithm was used to map the short read sequences to the NCBI 37 mouse genome assembly. **(a) **The percentage of reads that map to chromosome 17, other mouse chromosomes, or not at all to the C57BL/6J reference assembly. **(b) **The actual average sequence depth over chromosome 17 after duplicate sequence reads have been removed, plotted against the nominal depth if all reads were unique and mapped to chromosome 17. **(c) **The number of contiguous blocks of sequence, defined as a stretch of sequence where all bases have non-zero sequencing depth over them, plotted against nominal depth (see above).

**Figure 2:**
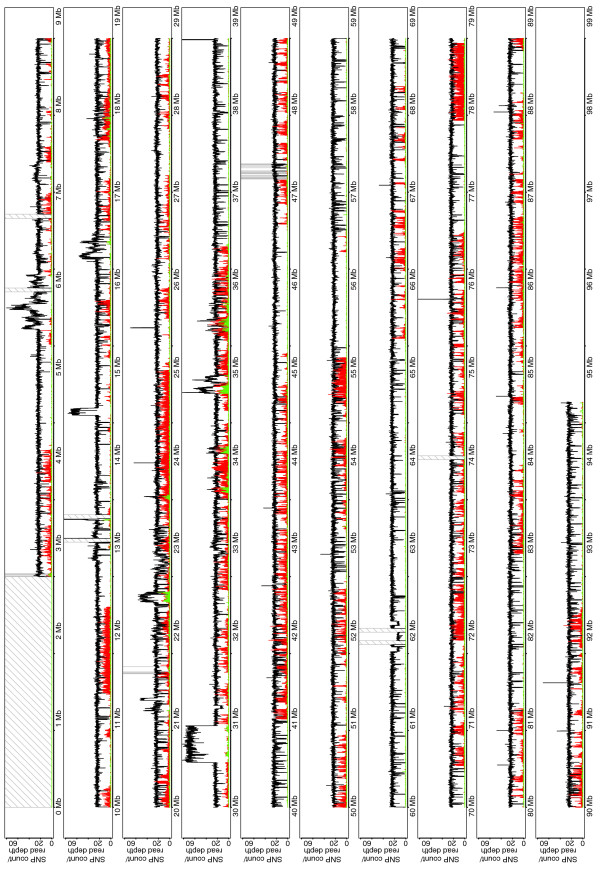
Read depth and SNP density for A/J on chromosome 17. Plot shows depth (black) and density of homozygous (red) and heterozygous (green) SNPs compared to the C57BL/6J reference along chromosome 17. Gray or hashed bars are gaps in the reference assembly.

We prepared primary mouse embryonic fibroblast cultures from E14.5 A/J and CAST/Ei embryos derived from pedigreed stock housed at MRC-Harwell. Genetic purity was confirmed by genotyping the embryos and their parents with a panel of polymorphic markers. Exponentially growing cultures were arrested in metaphase and chromosome 17 isolated by flow sorting [17]. A/J chromosome preparations were sheared to 150 to 200 bp in size and used to generate a library for paired-end sequencing on the Illumina Genome Analyzer platform [18]. In total, 10 lanes of sequencing was performed, producing 112,046,098 reads (Table 1). Reads containing uncallable bases were removed and the sequence was mapped to the C57BL/6J reference genome (version 37) using the Mapping and Assembly with Quality (MAQ) algorithm [19]. We successfully mapped 91.9% of sequence reads to the reference genome, 67.3% to chromosome 17 (Table 1 and Figure 1a). Reads mapping to chromosomes other than 17 may be the result of contamination of the chromosomal preparation with DNA from other chromosomes, the presence of sequence repeated on both chromosome 17 and another chromosome, or due to the translocation of sequence from chromosome 17 to another chromosome in the A/J genome. Duplicate reads, where the outer genomic co-ordinates of read pairs were identical, were removed, yielding an average A/J sequence depth over chromosome 17 of 22× (calculated as the number of reads mapping to the chromosome multiplied by the length of the reads and divided by the length of the chromosome). Figure 2 shows the mapping depth across all points on the chromosome.

**Table 1:** Sequencing statistics

	A/J	CAST/Ei
Number of Illumina lanes	10	10
Total number of bases (bp)	3,828,787,991	5,476,237,877
Total number of reads	112,046,098	173,021,348
% Mapped (in pairs)	91.9% (87.9%)	88.3% (80.0%)
% Duplicate read pairs	22.1%	18.2%
Modal insert size (± 1 SD)	130 (± 24) bp	118 (± 12) bp

SD, standard deviation.

**Figure 3:**
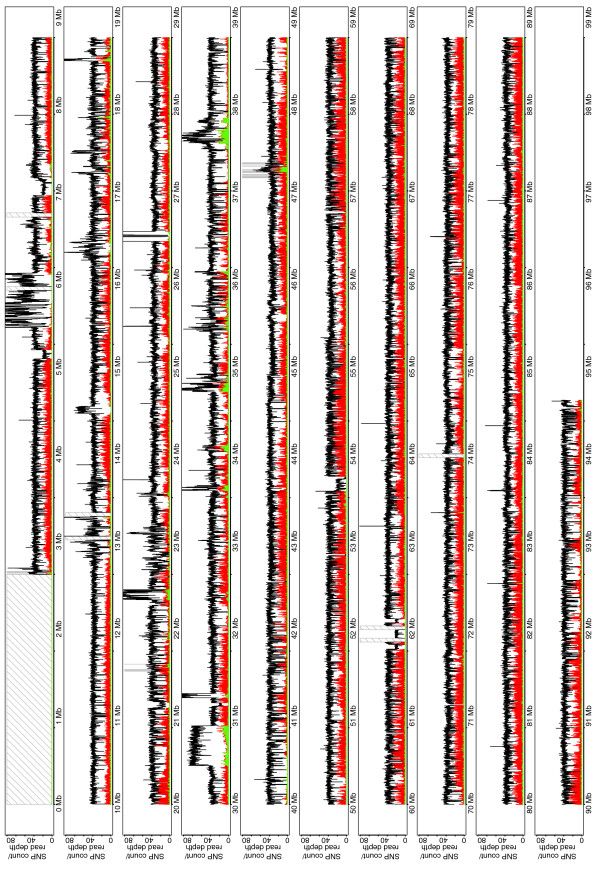
Read depth and SNP density for CAST/Ei on chromosome 17. Plot shows depth (black) and density of homozygous (red) and heterozygous (green) SNPs compared to the C57BL/6J reference along chromosome 17. Gray or hashed bars are gaps in the reference assembly.

We examined the effect of generating increasing amounts of sequence from the same library by mapping an increasing number of lanes of sequence data to the reference genome. Figure 1b shows that the coverage over chromosome 17 is directly proportional to the amount of sequence mapped, indicating that we did not exhaust the diversity present in our A/J chromosome 17 library. Figure 1c illustrates that the number of blocks, a stretch of genome where no base has a zero sequence depth over it, drops as the amount of sequence increases, but at 18× coverage (4 lanes) this figure asymptotes, suggesting that further sequencing of this library would not greatly increase the proportion of the genome covered. We defined the sequenceable fraction of mouse chromosome 17, using our approach, to be 98.5%. This figure may be restrained, in part, by the complexity of the library, or by the fact that the library had a defined insert size, but is also likely to reflect the underlying differences between the A/J genome and that of C57BL/6J. To sequence CAST/Ei chromosome 17, we employed a similar approach yielding 173,021,348 reads from 10 lanes of sequencing (Table 1). Figure 1a shows that a similar proportion of reads map to chromosome 17 as for A/J. There is a small increase in the number of reads mapping to other chromosomes in CAST/Ei. This is to be expected since *M. m. castaneus *is more evolutionarily distant from C57BL/6J, which, like A/J, is a classical inbred mouse strain and largely *M. m. domesticus *derived [8,10]. Relationships between the amount of sequence, coverage, and the number of blocks for CAST/Ei were similar to those observed for A/J. Figure 3 shows the mapping depth at each point on the chromosome.

### Nucleotide variant calling from A/J and CAST/Ei short read sequence data

We used the MAQ algorithm to identify candidate SNPs between A/J, CAST/Ei and the reference C57BL/6J sequence (NCBI m37). We then applied the MAQ SNP filter to filter the candidate SNPs on the basis of quality and indicators of repetitive sequence. In total, 181,442 homozygous and 10,209 heterozygous SNPs passed the filter and were identified between the A/J sequence and the reference C57BL/6J sequence of chromosome 17. For CAST/Ei, 657,558 homozygous and 34,173 heterozygous SNPs were identified on comparison to the reference and passed the MAQ SNP filter. The distribution of homozygous and heterozygous SNPs is shown in Figures 2 and 3. Importantly, heterozygous SNPs, which should not be present in the genome of a homozygous inbred mouse, cluster in regions with an increased mapping depth, suggesting that heterozygous SNPs mark the presence of segmentally duplicated sequences, of which one copy is mutated, rather than the presence of genuinely heterozygous genomic positions.

**Figure 4:**
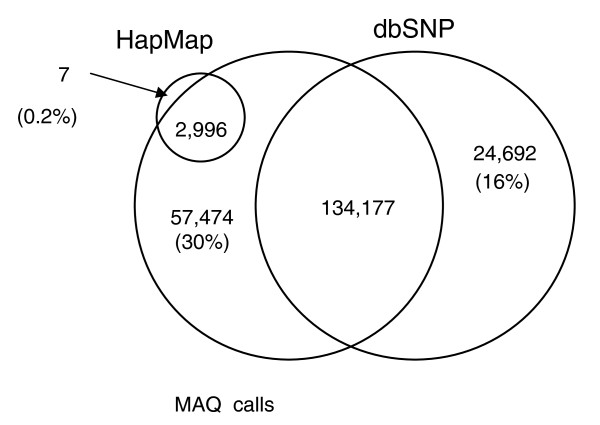
Analysis of SNPs found in A/J sequence. SNPs were called by the MAQ algorithm and then filtered using the MAQ SNP filter. An overlap of SNPs called by MAQ using the A/J Illumina data (MAQ Calls), those present in dbSNP, and those present in the 'Mouse HapMap'.

**Figure 5:**
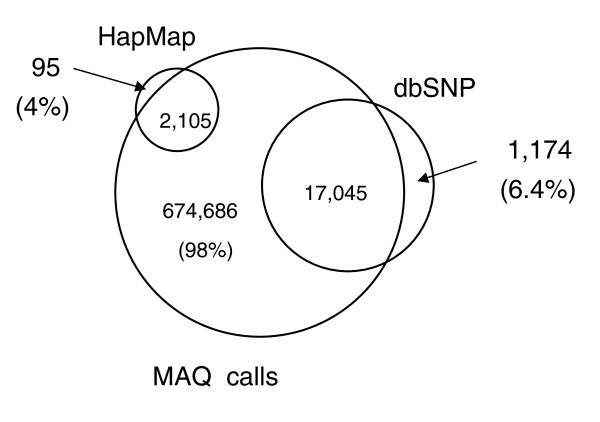
Analysis of SNPs found in CAST/Ei sequence. SNPs were called by the MAQ algorithm and then filtered using the MAQ SNP filter. The overlap between the SNPs called by MAQ using the CAST/Ei Illumina data (MAQ Calls), those present in dbSNP, and those present in the 'Mouse HapMap'.

To benchmark the success of SNP variant calling, we first compared SNPs called by MAQ with a collection of highly curated SNPs designated the 'Mouse HapMap' collection [20]. This set contains 3,003 positions on chromosome 17 that are polymorphic between A/J and C57BL/6J, of which all but 7 were identified correctly by MAQ, an accuracy of 99.77% (Figure 4). Importantly, these SNPs are derived from a collection used as part of a hybridization genotyping protocol and, as such, are in non-repetitive regions of the mouse genome. We next compared our MAQ SNP calls to a collection of 158,869 positions reported to be polymorphic between A/J and C57BL/6J in the dbSNP database (release 126). Of these SNPs, 134,177 (84%) were called by MAQ. This leaves 57,474 novel SNPs identified by MAQ. dbSNP A/J SNPs are largely derived from variant calling from the shotgun capillary read sequencing of A/J by Celera; thus, the increased accuracy of calling Mouse HapMap SNPs is likely to be due to the fact that the SNPs from the mouse HapMap are verified, and that they are easy to genotype SNPs, suggesting they are located in less repetitive regions of the genome. Figure 5 presents the same analysis for MAQ calls derived from the short read sequencing of CAST/Ei chromosome 17 where 96% of informative mouse HapMap SNPs and 94% of CAST/Ei SNPs in dbSNPs were called correctly. A far higher proportion of the SNPs in dbSNP between CAST/Ei and C57BL/6J are present in our sequencing data, although they represent a much smaller proportion of the SNPs called. Again, this is probably due to the fact that known SNPs between CAST/Ei and C57BL/6J are in easy to genotype regions of the genome. The novel SNPs identified here represent a greater than 35-fold increase in the number of known SNPs between CAST/Ei and C57BL/6J on chromosome 17, illustrating the power of this technology for nucleotide variant discovery.

**Figure 6:**
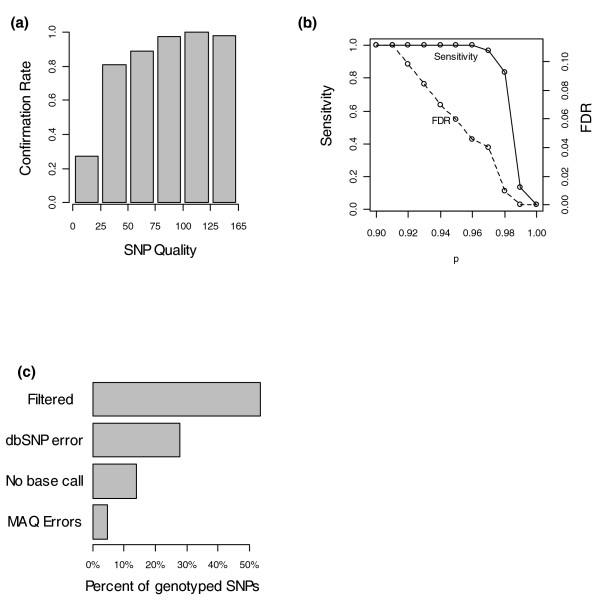
Quality control analysis of novel A/J SNPs. A sample of novel SNPs was genotyped using the Sequenom platform. **(a) **Plot shows the proportion of calls confirmed by genotyping for differing qualities of SNP. **(b) **Quality controlling SNPs on basis of mapping depth as well as quality. Confirmation data were used to calculate a score for each SNP based on quality and depth. Plot shows estimated sensitivity and false discovery rate (FDR) based on using different thresholds of p-score. **(c) **SNPs missed by MAQ. A sample of SNPs present in dbSNP but absent from the MAQ was were genotyped. The reason for the absence of each SNP is shown. 'Filtered' SNPs were called by MAQ but filtered out as being of low quality. 'dbSNP errors' are SNPs where our genotyping agrees with the MAQ call but not dbSNP. 'No base call SNPs' are SNPs for which MAQ did not make a base call (generally due to zero depth). MAQ errors are bases where our genotyping agreed with the dbSNP call.

In order to quality control our MAQ SNP calls, we divided the novel SNPs between A/J and C57BL/6J into 6 equal bins based on the MAQ quality score for each SNP and randomly selected 50 homozygous SNPs from each bin. We also selected 20 heterozygous SNPs with a high quality and average sequence depth. We reasoned that such SNPs are most likely to represent genuine heterozygous SNPs. These SNPs were genotyped using the Sequenom genotyping platform using DNA derived from the embryo from which chromosome 17 was isolated, the parents of this embryo, and two sibling embryos. In total, 199 of the homozygous and 10 of the heterozygous SNPs were successfully genotyped. Importantly, the allele called for each SNP was the same in all of the five genotyped samples, meaning that all the SNPs genotyped are likely to be homozygous. This lends support to the hypothesis that heterozygous SNPs called in the sequence data represent repetitive regions or denote structural variants rather than genuine heterozygous SNPs. In 88.4% of cases the allele called by the genotyping assay was the same as the allele called by MAQ from the sequence data. Figure 6a shows the proportion of novel SNPs that were confirmed by genotyping in each quality bin. The probability of confirming a SNP dramatically increases with qualities over 25. However, discarding all SNPs with qualities less than 50 would result in the loss of many genuine SNPs. In order to find a more sensitive method of quality controlling SNPs, we combined the quality score of the SNPs with the sequencing depth over them. For each SNP we calculated the proportion of SNPs in the genotyped sample with the same or higher depth and quality that were confirmed and used this to assign a p-score to each SNP. This allows a much more sensitive quality control of novel SNPs (Figure 6b). Taking the 46,676 novel homozygous SNPs with a p-score of 0.95 or greater only, we estimate a false discovery rate of 5.9% and with 99.97% of the genuine SNPs being called.

In order to discover why we had missed SNPs recorded in dbSNP, we choose 300 at random and genotyped them on the Sequenom platform. In total, we successfully genotyped 172 loci. In 27.9% of cases the allele called by the genotyping experiment agreed with our sequencing data, suggesting that in these cases the SNP recorded in dbSNP is not present in the A/J isolate we sequenced (Figure 6c). This figure was similar irrespective of the verification status of the SNP in dbSNP. Assuming that this is true of all SNPs present in dbSNP but absent from our sequencing data, we estimate our sensitivity at finding known SNPs to be 88.4%. Examining the loci where our genotyping suggested the allele specified in dbSNP was present in our sample, we found that in 53.5% of cases the correct base was called by MAQ, but was filtered out as being of low quality by the MAQ SNP filter. In 14.0% of cases no base was called by MAQ. This was generally due to no sequencing reads mapping to this location. In only 4.6% of cases did MAQ make an incorrect base call (Figure 6c).

In this study we have increased the number of known SNPs between A/J and C57BL/6J on the reference assembly of chromosome 17 by 29% (46,676) and with an estimated accuracy of 94.1% and a sensitivity of 88.2% using a p-score threshold of 0.95 (for the effects of different p-score thresholds see Additional data file 1). Although all genotyping was carried out in A/J samples using SNPs identified in A/J sequence, we expect that our performance in calling SNPs between CAST/Ei and C57BL/6J to be similar since an identical approach was used. In this way we identify 631,273 novel homozygous SNPs (increasing the number of known CAST/Ei SNPs on chromosome 17 35-fold), with a similar estimated accuracy and sensitivity (see Additional data file 2 for different p-score thresholds).

As well as SNPs, short insertion and deletion mutations (indels) can also be important in changing the function of the DNA sequence. We attempted to predict indels from the Illumina sequence for the two strains. In total, we predicted 32,564 indels in the A/J sequence when compared to the reference, and 97,784 indels between the reference and CAST/Ei. In order to assess the performance of our indel prediction, we compared these indels to those predicted from the 2.5× shotgun capillary sequence of the A/J genome generated by Celera. Of the 24,142 indels predicted from the capillary sequence, we found perfect matches for 4,348 in the short read sequence. In 7,351 cases an indel of the correct type (that is, insertion or deletion) was found in the right place, but the predicted base change was different. In 857 cases we found indels at the correct position, but of the incorrect type (that is, insertion where their should be a deletion and vice versa). A Venn diagram of this analysis is provided in Additional data file 3. This indicates that although indels can be predicted from short-read sequence, the quality of such calls cannot be assured to the same extent as the SNP calls.

### Identification of deletions and copy number variants

It is becoming increasingly evident that structural variation between strains and individuals is more widespread than had been previously appreciated [21-23]. We examined structural variation using two approaches. Firstly, we used information on the mapping of pairs of reads to identify regions that were potentially deleted between A/J or CAST/Ei when compared to the reference. Secondly, we used mapping depth and density of heterozygous SNPs to identify regions of increased or decreased copy number.

**Figure 7:**
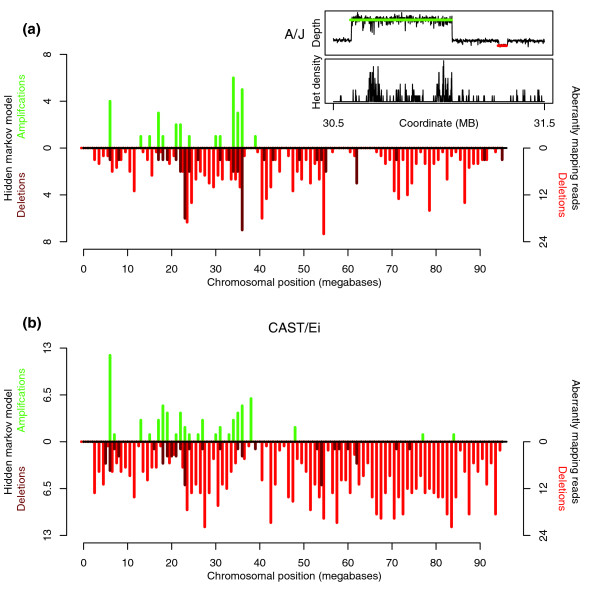
Structural variants on chromosome 17. Copy number variants (CNVs) were called in the sequence using a hidden Markov model (HMM), using both depth information and density of heterozygous SNPs (for amplifications). Deletions were called using aberrantly mapping pairs of reads. **(a) **Density of CNVs and deletions across chromosome 17 of A/J. CNVs called by the HMM are shown in dark red/green on the left-hand axis. Deletions called from aberrantly mapping read pairs are shown in bright red on the right hand axis. **(b) **As (a) but for CAST/Ei chromosome 17. Cut-out shows an example of output from the HMM for a region with two amplifications and a loss.

To call deletions in the sequence in comparison to the reference sequence, we identified clusters of reads for which the two ends of the pairs map further apart from each other than would be expected. We identified 864 cases in the A/J sequence where two or more overlapping pairs of reads were separated by more than 4 standard deviations from the average length of paired-end sequenced molecules. In order to quality control these calls, we selected 52 cases where these regions overlapped with exons in the reference sequence and sequenced the region in C57BL/6J and A/J genomic DNA samples using a traditional PCR and capillary sequencing approach. In 92% of cases where the predicted deleted region is longer than 450 bp or found in 4 or more read pairs, we found a deletion in the A/J sequence compared to the reference sequence. In one of these cases we also found a deletion in the C57BL/6J sequence, suggesting that the genome of the C57BL/6J individual we sequenced is different from the reference sequence, or there is an error in the reference genome. We found a deletion in only 2.5% of cases where the predicted deleted region is both less than 450 bp and found in fewer than 4 read pairs. Therefore, we filtered our predicted deletions for those either larger than 450 bp or found in 4 or more deletion pairs. Thus, we predicted 416 deletions on chromosome 17 of A/J compared to the reference sequence (Figure 7a; Additional data file 4). We predicted 776 deletions on chromosome 17 of CAST/Ei compared to the reference in the same way (Figure 7b; Additional data file 5).

In addition to calling deletions using aberrantly mapping read pairs, we also identified regions of increased or decreased copy number. The central idea behind our copy number variation (CNV) detection algorithm is that the alignment of reads from regions with copy number gains (with respect to the reference genome) will be 'collapsed' to a single location on the reference genome. The effect of this will be two-fold. First, the sequence depth of the location on the reference genome will be increased by an integral amount corresponding to the relative number of copies that exist in the individual. Second, any base-pair differences between the copied regions will appear to contain heterozygous SNPs. This fact is crucial to our model as laboratory strains of mice, and indeed wild-derived strains, are inbred to be effectively homozygous at every position in the genome [6,8,24], hence any apparent heterozygous SNPs that are not the result of sequencing or mapping errors are actually from collapsed regions. The read alignments of regions with copy number losses will be distributed over the corresponding copies in the reference genome, and hence the reference regions will have lower sequence depth (with the important distinction that there should not be a heterozygous SNP signal). We developed a hidden Markov model (HMM) to exploit these facts and detect copy number gain, or loss (JT Simpson *et al*., submitted).

This HMM identified 31 copy number gains and 43 copy number losses on A/J chromosome 17 (average length 46.83 kb and 17.9 kb, respectively) and 66 copy number gains and 48 copy number losses on CAST/Ei chromosome 17 (average length 35.0 kb and 26.0 kb respectively). The distribution of these CNVs on chromosome 17 is shown in Figure 7. A complete list of regions is available in Additional data file 6. In order to assess the success of our HMM for identifying CNVs, we compared the CNVs called to those called by She et al. [23] in an extensive microarray-based comparative genomic hybridization (aCGH) study [23]. For A/J, we identified 71% (0.64 Mb of sequence) of the copy number gains identified by aCGH. For CAST/Ei, 91% (0.87 Mb) of the aCGH copy number gains were identified by our model. In both strains the regions of copy number loss called by our algorithm and aCGH differed widely (23% concordance for A/J and 38% for CAST/Ei) owing to the relative difficulty of calling CNV losses compared to gains. For many CNVs we were able to more accurately refine the boundary of the CNV using the sequencing data. We also predict 66 new copy number gains that had not been described previously (22 for A/J and 44 for CAST/Ei), and 71 novel copy number losses (35 for A/J and 36 for CAST/Ei).

Together these data show that we can effectively call both small deletions and larger structural variants from paired end sequence data by exploiting read-pair and mapping depth information and the distribution of heterozygous SNPs.

### Coding differences between genes on chromosome 17

Coding variation is thought to contribute significantly to the phenotypic differences between mouse strains. In total, we identified 2,386 SNP differences within coding sequence between A/J and C57BL/6J (Additional data file 7) and 5,959 SNP differences between CAST/Ei and C57BL/6J (Additional data file 8), of which 1,100 and 2,574 positions, respectively, represented non-synonymous coding changes, or changed a splice site. The SNP differences most likely to have a measurable effect on the protein product of a gene are changes (either gain or loss) to stop codons. Of the coding SNPs we identified, 10 genes in A/J and 25 genes in CAST/Ei were predicted to carry either gains or losses of stop codons (Table 2); one gene, 9130008F23Rik (ENSMUSG00000054951), was found to carry two stop codons in CAST/Ei. Interestingly, in three cases (the gain of a stop-codon at codon 70 of *Rpp21*, and loss of stops in both *Rmcs2/H2-Ab1 *and ENSMUSG00000073373), both the A/J and the CAST/Ei sequences contain the same changes compared to C57BL/6J, suggesting that the A/J and CAST/Ei alleles may be derived from the ancestral alleles of these genes.

**Table 2:** Stop codons gained and lost relative to reference

Gene	Description	Codon
A/J		
Stops gained		
*Abca3*	ATP-binding cassette sub-family A member 3	1069/1704
*Lmf1*	Transmembrane protein 12	182/188
ENSMUSG00000073427	Adult male hypothalamus cDNA	109/132
ENSMUSG00000002791		109/311
*EG630249*	Butyrophilin-like 7	570/580
*H2-T23*	H-2 class I histocompatibility antigen, D-37 alpha chain precursor	218/357
*Rpp21*	Ribonuclease P protein subunit p21	70/71
ENSMUSG00000073395	Adult male epididymis cDNA	158/163
Stops lost		
*Rmcs2*	Response to metastatic cancers 2 gene	263/263
ENSMUSG00000073373	12 days embryo female mullerian duct includes surrounding region cDNA	42/42
		
CAST/Ei		
Stops gained		
*Pabpc3*	Poly(A) binding protein, cytoplasmic 3 gene	631/644
ENSMUSG00000073464	Putative uncharacterized protein	107/140
*Smok2a*	Sperm motility kinase 2B gene	96/505
*BC052484*	Mesothelin-like gene	673/686
*Uhrf1 bp1*	UHRF1 (ICBP90) binding protein 1 gene	89/93
*C920016K16Rik*	RIKEN cDNA C920016K16 gene	13/468
ENSMUSG00000067203		3/385
*2610110G12Rik*	RIKEN cDNA 2610110G12 gene	398/399
*H2-T9*	Histocompatibility 2, T region locus 22 gene	126/380
*Rpp21*	Ribonuclease P 21 subunit (human) gene	70/72
ENSMUSG00000044538		305/320
*Olfr138*	Olfactory receptor 138 gene	203/313
9130008F23Rik ENSMUSG00000054951	RIKEN cDNA 9130008F23 gene	55+106/182
*Trem2*	Triggering receptor expressed on myeloid cells 2 gene	148/250
*EG328839*	Predicted gene, EG328839	13/115
*Yipf4*	Yip1 domain family, member 4 gene	30/290
ENSMUSG00000079336		3/13
ENSMUSG00000066938	Putative uncharacterized protein fragment	94/108
*Thada*	Thyroid adenoma associated gene	1791/1950
ENSMUSG00000071036	Putative uncharacterized protein MCG125396	53/120
Stops lost		
*Rmcs2*	Response to metastatic cancers 2 gene	263/263
*H2-Bl*	Histocompatibility 2, blastocyst gene	263/263
*Foxn2*	Forkhead box N2 gene	212/212
ENSMUSG00000073373	Putative uncharacterized protein	42/42

We also predicted coding sequences that are affected by structural rearrangements. In principle, a structural rearrangement can overlap with a coding exon in four ways: the borders of the rearrangement can completely contain an exon, the rearrangement can be completely contained within an exon, or the overlap can affect only the start or end of an exon. Given that the borders of deletions called from aberrantly mapping read pairs have a resolution of around 50 bp and those of CNVs from the HMM a resolution of around 10 kb, we are only confident that a deletion overlaps with an exon where the exon is completely contained within the deletion or the overlap is at least 50 bp. Similarly, we are only confident of the overlap between a CNV and an exon where the exon is completely contained within the CNV region. A higher number of protein coding sequences are potentially affected by structural rearrangements than changes in stop codons. There were nine genes predicted to have exons overlapping with deletions predicted from A/J sequence and ten genes from CAST/Ei sequence (Table 3). Of these, all those in the A/J sequence have been confirmed by traditional capillary sequencing (see above). A total of 53 and 54 genes have exons within regions identified as having copy number gain by the CNV HMM in A/J and CAST/Ei sequences, respectively (427 and 520 ensembl exons), while 15 and 16 genes (66 and 95 ensembl exons) have exons contained within regions identified as having copy number loss (Additional data file 9). The effect of a copy number gain may be less than that of complete exon deletion, so it is perhaps not surprising that a larger number of exons are amplified than deleted, especially as in the majority of cases of exon amplification, all exons of a gene are predicted to be amplified.

**Table 3:** Genes and transcripts affected by deletions predicted from aberrantly mapping sequence pairs

Gene	Transcript	Strain	Exons
*1700001C19Rik*	ENSMUST00000073143	CAST/Ei	1
*Alk*	ENSMUST00000086639	A/J	19
*Capn13*	ENSMUST00000095208	CAST/Ei	7
*EN*	ENSMUST00000052440	CAST/Ei	1, 2
	ENSMUST00000077420	CAST/Ei	1
	ENSMUST00000079363	CAST/Ei	1
	ENSMUST00000086423	CAST/Ei	1
ENSMUSG00000060087	ENSMUST00000077584	A/J	1, 2
*Galnt14*	ENSMUST00000024858	CAST/Ei	15
*Gfer*	ENSMUST00000046839	A/J	3
	ENSMUST00000046839	CAST/Ei	3
*mmu-mir-692-1*	ENSMUST00000102263	A/J	1
	ENSMUST00000102263	CAST/Ei	1
*Olfr55*	ENSMUST00000112168	CAST/Ei	1
*Prss28*	ENSMUST00000015267	A/J	1-7
*Rpl35a*	ENSMUST00000087940	A/J	1
	ENSMUST00000087940	CAST/Ei	1
*Tbc1d24*	ENSMUST00000040474	A/J	9
	ENSMUST00000040474	CAST/Ei	9
	ENSMUST00000097376	CAST/Ei	10
*U6*	ENSMUST00000102026	A/J	1
*V1rf3*	ENSMUST00000077301	CAST/Ei	1
*Wiz*	ENSMUST00000087699	A/J	4

One important region on mouse chromosome 17 is the MHC, which codes for a large number of genes involved in immunity [11]. A large number of genes affected by coding changes are associated with the MHC complex (Table 2) and more genes annotated as members of the MHC complex under the Gene Ontology are predicted to be in CNV regions (Table 4). A significant enrichment of MHC genes amongst those genes with such changes (*P *= 2.23 × 10^-9 ^for A/J, and *P *= 3.55 × 10^-5 ^for CAST/Ei, Fisher's exact test), indicates that this region is significantly more variable than other regions of the chromosome.

**Table 4:** Genes and transcripts associated with the MHC complex affected by CNVs as predicted by a hidden Markov model

Gene	Transcript	Type	Strain	Exons
*2410017I17Rik*	ENSMUST00000090537	Gain	A/J	1- 7
	ENSMUST00000090537	Loss	CAST/Ei	1-6
*CR974462.5-201*	ENSMUST00000087244	Gain	CAST/Ei	1-5
*H2-D1*	ENSMUST00000087173	Gain	A/J	1-8
	ENSMUST00000078966	Gain	A/J	1-7
	ENSMUST00000087173	Gain	CAST/Ei	1-8
	ENSMUST00000078966	Gain	CAST/Ei	1-7
*H2-DMb2*	ENSMUST00000114232	Gain	A/J	1-6
*H2-K1*	ENSMUST00000114311	Gain	A/J	1-7
	ENSMUST00000087189	Gain	A/J	1-9
	ENSMUST00000025181	Gain	A/J	1-8
	ENSMUST00000046131	Gain	A/J	1-7
	ENSMUST00000114311	Gain	CAST/Ei	1-7
	ENSMUST00000087189	Gain	CAST/Ei	1-9
	ENSMUST00000025181	Gain	CAST/Ei	1-8
	ENSMUST00000046131	Gain	CAST/Ei	1-7
*H2-M10.5*	ENSMUST00000041531	Gain	A/J	1
*H2-M10.6*	ENSMUST00000041398	Gain	CAST/Ei	1, 2
*H2-Q1*	ENSMUST00000105041	Gain	A/J	1-3
	ENSMUST00000073208	Gain	A/J	1-8
	ENSMUST00000074806	Gain	A/J	1-7
	ENSMUST00000078205	Loss	A/J	1-8
	ENSMUST00000113887	Loss	A/J	6, 7
	ENSMUST00000081435	Loss	A/J	5, 6
	ENSMUST00000078205	Gain	CAST/Ei	1-3
	ENSMUST00000105041	Gain	CAST/Ei	2
	ENSMUST00000073208	Gain	CAST/Ei	4-8
	ENSMUST00000113887	Gain	CAST/Ei	1-4
	ENSMUST00000081435	Gain	CAST/Ei	1-3
	ENSMUST00000074806	Gain	CAST/Ei	1-7
	ENSMUST00000078205	Loss	CAST/Ei	1-8
	ENSMUST00000113887	Loss	CAST/Ei	6, 7
	ENSMUST00000081435	Loss	CAST/Ei	5, 6
*H2-Q10*	ENSMUST00000056774	Gain	A/J	1-4
	ENSMUST00000068291	Gain	A/J	1-3
	ENSMUST00000040279	Gain	A/J	1-3
	ENSMUST00000056774	Gain	CAST/Ei	1-4
	ENSMUST00000068291	Gain	CAST/Ei	1-3
	ENSMUST00000040279	Gain	CAST/Ei	1-3
*H2-Q2*	ENSMUST00000040240	Gain	A/J	1-6
*H2-Q7*	ENSMUST00000071951	Gain	A/J	7
*H2-Q7*	ENSMUST00000076256	Gain	A/J	7, 8
*H2-T10*	ENSMUST00000074201	Loss	A/J	1-10
*H2-T18*	ENSMUST00000025312	Gain	A/J	1-6
	ENSMUST00000095300	Gain	A/J	1-4
	ENSMUST00000097329	Gain	A/J	1, 2
	ENSMUST00000113714	Gain	A/J	1-4
	ENSMUST00000102675	Gain	A/J	1-6
	ENSMUST00000025312	Gain	CAST/Ei	1-6
	ENSMUST00000095300	Gain	CAST/Ei	1-4
	ENSMUST00000097329	Gain	CAST/Ei	1, 2
	ENSMUST00000113714	Gain	CAST/Ei	1-4
	ENSMUST00000102675	Gain	CAST/Ei	1-6
*H2-T9*	ENSMUST00000058801	Loss	A/J	1-9
	ENSMUST00000077960	Loss	A/J	1-10
	ENSMUST00000080015	Loss	A/J	1-9
*NM_001025208.1*	ENSMUST00000064686	Gain	A/J	1-9
	ENSMUST00000064686	Gain	CAST/Ei	1-9

### *De novo *assembly of mouse chromosome 17 from A/J and CAST/Ei

The analysis described so far is based on mapping reads to the C57BL/6J reference sequence. This approach, while informative of SNPs and structural variation, does not tell us about additional sequence present in A/J or CAST/Ei but not in the reference. To assess this we explored *de novo *assemblies of the reads. For CAST/Ei chromosome 17, we used a novel de Bruijn graph based algorithm called Fuzzypath to generate short sequence contigs from the Illumina reads of this chromosome and then to present these to the Phusion assembler [25]. A full description of this algorithm is available from the Fuzzypath website [26].

The resulting assembly of the chromosome totaled 76 Mb in length with an N50 of 2,315 bp (N50 is the length of the shortest contig such that the sum of contigs of equal length or longer is at least 50% of the total length of all contigs).

We compared the performance of FuzzyPath on these data with two other assembly algorithms designed for generating *de novo *assemblies from short read sequencing - the widely used Velvet algorithm [27] and the newly released algorithm AbySS [28]. A kmer length of 24 was used for each algoithm but all other parameters were default. FuzzyPath outperformed Velvet by a wide margin on all the metrics we considered (Table 5), particularly N50. We were unable to use the latest version of Velvet as the 192 Gb of RAM on our machine was insufficient. The difference between FuzzyPath and ABySS was similar for assembled bases and contig coverage, but the N50 for the FuzzyPath was more than twice that of the ABySS assembly.

**Table 5:** Assembly statistics

Assembler	Sequencing	Strains	Insert size	Read length (bp)	Number of reads	Raw read coverage	Assembled bases (Mb)	Contig coverage	Contig N50
FuzzyPath	Illumina	CAST/Ei	200 bp	36	173 million	65×	76.03	80.0%	2,315
Velvet*	Illumina	CAST/Ei	200 bp	36	173 million	65×	58.09	61.15%	391
ABySS	Illumina	CAST/Ei	200 bp	36	173 million	65×	72.80	76.63%	1,022
FuzzyPath	Illumina	A/J	200 bp	36	112 million	42×	55.87	58.8%	959
Phusion	Capillary	A/J	3-5 kb	400-900	357,100	2.5×	81.2	85.5%	3,377
FuzzyPath	Hybrid	A/J^†^	Hybrid^†^	Hybrid^†^	Hybrid^†^	Hybrid^†^	85.54	90.0%	5,793

*Version 0.4. We also tried the latest version 0.7, which had been much improved in terms of contig length, but ran out of memory on a computer with 192 Gb RAM. ^†^Assembly incorporating the A/J Illumina reads and Celera capillary reads.

For A/J the read coverage was 22×, significantly lower than for CAST/Ei and the read quality was also lower, and we were only able to obtain an assembly of 55.87 Mb with an N50 of 959 bp. However, 2.5× whole genome shotgun capillary read coverage of A/J is available from sequence generated by Celera. Assembling this sequence on its own generates a better assembly than the short-read sequencing, demonstrating the difficulties of assembling short reads compared to long read sequence (Table 5). One of the most important novel features of FuzzyPath is its ability to combine both short and long reads. Thus, we were able to combine the capillary sequence data of A/J chromosome 17 with the Illumina sequence data of this chromosome to generate a hybrid assembly (see Materials and methods). The resulting *de novo *assembly showed a slight improvement of contig coverage and assembled bases and a significant increase in N50 (from 3,377 to 5,793). Table 5 lists the statistics for the assemblies that were generated. We could not be align 144 kb of the A/J assembly generated using both the Celera reads and Illumina data and 170 kb of the short read CAST/Ei assembly anywhere on the entire C57BL/6J reference genome assembly (>95% match); these therefore represent 'novel sequence'. Analysis of this sequence revealed that some of it could be aligned to sequence in the NCBI trace archive derived from mouse strains other than the reference, some of it aligned to the rat genome assembly, and some of the contigs appeared to be novel 'repeat-like' sequence possibly of telomeric or centromeric origin. Unaligned contigs are available for download from [29].

### Identification of candidate genes that regulate liver triglyceride levels on chromosome 17

**Figure 8:**
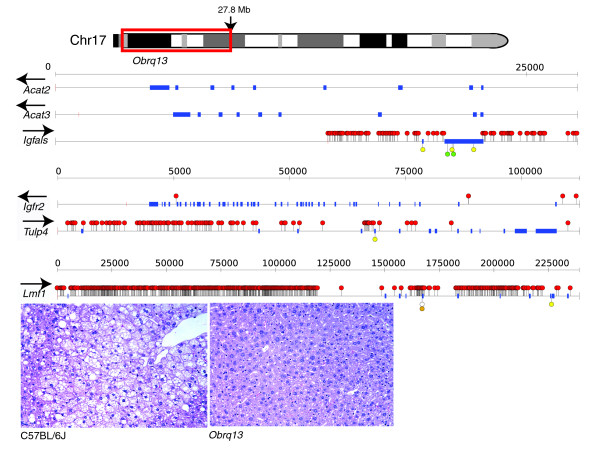
Analysis of SNPs in candidate genes within the *Obrq13 *QTL region of mouse chromosome 17 that has a protective effect on liver triglyceride levels. Shown is an example of histology of the liver of a C57BL/6J male and a consomic mouse carrying the *Obrq13 *region from A/J, which has a protective effect on the accumulation of liver triglycerides when animals are placed on a high fat diet [16]. Using the sequence of A/J chromosome 17, we called SNPs against the reference C57BL/6J genome and positioned them in candidate genes within the *Obrq13 *region. Non-coding SNPs are shown as red circles, non-synonymous SNPs are shown in green, synonymous SNPs are shown in yellow while the truncating and essential splice site SNPs found in a transcript of *Lmf1 *are shown as an open circle and an orange circle, respectively. The orientation of each gene relative to the forward strain is shown above the gene name as an arrow and genes are grouped together based on size (a scale is shown above each group of genes). Genes are displayed with 5 kb of genomic sequence 5' and 3'.

An extensive chromosome 17 substitution strain panel was generated by interbreeding C57BL/6J and A/J mice [15] and was used to identifying several regions that contain loci that regulate liver triglyceride levels [16]. Importantly, a locus between the centromere and 27.8 Mb was identified that has a protective effect on high liver triglyceride levels since male consomic mice carrying this interval from A/J (*Obrq13*) were found to have lower liver triglyceride levels when placed on a high fat diet compared to C57BL/6J controls [16] (Figure 8). Within the *Obrq13 *interval there are six strong candidate genes implicated in lipid metabolism. These are the insulin-like growth factor II receptor (*Igf2r*), insulin-like growth factor binding protein (*Igfals*), acetyl-coenzyme A acetyl transferase 2 (*Acat2*) and *Acat3*, the tubby-like protein 4 (*Tulp4*), and the lipase maturation factor (*Lmf1*). Using the sequence of A/J chromosome 17, we were able to profile all of the coding and non-coding variants in and around these genes (Figure 8). We found that the genes *Acat2 *and *Acat3 *carried no intergenic or coding SNPs; in fact, we found no SNPs 5 kb upstream or downstream of these genes. While it is possible that a mutation in a far upstream regulatory element may control the function of these genes, they seem the less likely candidate genes within the interval. Similarly, the gene *Igf2r *contains no coding SNPs and only four SNPs are found within this gene, all of them intronic and none in the untranslated regions. The three genes carrying the highest number of polymorphisms were *Lmf1*, *Igfals *and *Tulp4*, which all carry a significant number of proximal promoter variants. Notably, *Tulp4 *was predicted to carry several CNVs (Additional data file 9). Intriguingly, *Lmf1 *was found to contain a splice site SNP, and a stop codon in a splice variant of the gene (Figure 8). Importantly, mice with loss of function mutations in *Lmf1 *develop severe hypertriglyceridemia [30]. Thus, by having the sequence of A/J chromosome 17 we have been able to rank three genes within the interval (*Igfals*, *Tulp4 *and *Lmf1*) as the most worthy genes for follow up functional analysis.

## Discussion

Here we present the first new-technology sequencing of a murine chromosome. This gives a glimpse of what will be achievable from the larger project we are undertaking to sequence the entire genomes of 17 mouse strains [31]. We show that short-read sequencing of the mouse genome on the Illumina platform is an efficient way of discovering both nucleotide and structural variation and that it is possible to use these data to generate chromosomal assemblies, which provide a valuable substrate for comparative and mutational analysis.

### Nucleotide variant discovery

Using MAQ, we called 181,442 homozygous and 10,209 heterozygous SNPs by comparing A/J chromosome 17 to the reference C57BL/6J assembly. Similarly, we identified 657,558 homozygous and 34,173 heterozygous SNPs by sequencing CAST/Ei chromosome 17. To the best of our knowledge this is the first report to assess the accuracy of the novel SNP calling. Previous studies have assessed the accuracy of SNP calling by comparison to known variant sites represented on genotyping arrays. These easy to type sites are analogous to our mouse HapMap SNP set, where we found a similarly high concordance. The accuracy of novel SNP calling is slightly lower, showing that it is possible that these SNPs are in harder to sequence and genotype regions of the genome. However, we have demonstrated that by carefully assessing the quality of each predicted SNP, this accuracy can be increased with only a small loss of sensitivity. Homozygous A/J SNPs are distributed in blocks along the chromosome (Figure 2) delineating conserved haplotypes between A/J and C57BL/6J. In contrast, CAST/Ei SNPs are evenly distributed along the chromosome. There were, however, several discrete regions towards the telomeric end of the chromosome that are SNP-poor and may thus represent *M. m. castaneus*-derived regions of the C57BL/6J genome. Since all classical and wild-derived mouse strains are inbred and their genomes fixed, they are homozygous at all loci. Thus, the identification of heterozygous SNPs is likely to denote regions of segmental duplication between the strains. Importantly, we show that the vast majority of these heterozygous variants fall within regions where there was an increase in the density of mapped sequenced reads, suggesting that the copy number of these regions varies between the strains. Fortunately, because we were working with inbred mouse strains we could disambiguate this pattern. Determining if a SNP is truly heterozygous in an outbred population or indeed in humans will be considerably more challenging. Importantly, we found that the accuracy of calling SNPs varies significantly. SNP calling in repetitive regions of chromosome 17 was less sensitive than in non- repetitive regions owing largely to our ability to uniquely place read pairs, and the resultant coverage of reads obtainable in these regions. In this experiment flow sorted chromosomal DNA was a limiting reagent and after having sorted chromosome 17 from over 10^8 ^cells we had only 300 ng of DNA. Genomic DNA will not, however, be a limiting reagent when sequencing entire mouse genomes, making it possible to generate multiple Illumina libraries with different insert sizes, which, together with longer sequence reads, should facilitate the sequencing of repetitive and complex regions. Importantly, we identified 29 nucleotide variants in 28 genes in A/J and CAST/Ei that are predicted to be truncating loss of function alleles. Given that mice are homozygous at every loci along chromosome 17, these are effectively null alleles. These truncated genes may include those that, from the point of view of the animals' development and reproduction, are non-essential or neutral mutations. Others may be mutations that have been selected for during the derivation of the strains. Several mutations, including those in the genes *Rpp21*, *Rmcs2 *and ENSMUSG00000073373, are present in A/J and CAST/Ei but not in the reference C57BL/6J genome (Table 2). This is interesting since it may represent an example of how these strains have diverged. Indeed, it will be interesting to genotype these variants in a larger panel of strains to determine the origin of these alleles.

Although expected to be less frequent than SNP mutations, indels are expected to have a large effect on gene function. However, we failed to be able to accurately confirm our indel predictions, with a concordance rate of only 18% between indels predicted from capillary sequence and short-read sequence (rising to 52% if only the position of indels, and not their content, is considered). It is not known if this disagreement is due to differences in the algorithms used to identify the mutations or due to a more fundamental difference in the data. Indel prediction from short-read sequence is clearly an area that would benefit from future attention.

### Copy number variation

We exploited two approaches for the identification of structural variants between C57BL/6J and A/J and CAST/Ei chromosome 17. The first involved using read pair information to identify aberrantly mapped reads defining regions where there has been a deletion relative to the reference genome. We identified 416 variants on A/J chromosome 17 and 776 on CAST/Ei chromosome 17 that are collectively predicted to affect 15 genes (Figure 7, Table 3). All these variants are novel. The second approach exploits the observation that heterozygous SNPs cluster together in regions where there are changes in the density of reads mapping to a location. Using a novel HMM-based algorithm that incorporates the density of heterozygous SNPs and read depth, we identified 74% to 91% of the copy number gains described in a previous high-resolution survey of CNV in the mouse genome and in many cases we were able to use our data to refine the resolution of these CNVs. We also predict 43 new variants that had not been described previously (Figure 7). These CNVs affect 82 genes (Additional data file 9). That we found a higher proportion of known CNV gains compared to losses demonstrates the value of using the extra information provided by the density of heterozygous SNPs for identifying gains.

### *De novo *chromosome assembly

To fully realize the goal of being able to interrogate the genomes of different mouse strains, it is desirable to generate *de novo *assemblies of each strain. We employed a new algorithm called 'Fuzzypath' to assemble our short read sequences of A/J and CAST/Ei chromosome 17. Fuzzypath exploits a de Bruijn graph algorithm [32] to build assembly trees and then ultimately contigs and outperformed other commonly used *de novo *assembly algorithms (Table 5). Remembering that the sequence of mouse chromosome 17 from A/J and CAST/Ei were derived from a single 150 to 200 bp paired-end Illumina library, we were able to obtain respectable N50 contig lengths and for A/J we were able to use Fuzzypath to generate a hybrid assembly using capillary shotgun reads that was significantly better than assemblies generated purely from short or longer read sequences individually (Table 5). The difference in N50 between the purely short-read assemblies and that of the hybrid assembly demonstrates the value of such hybrid methods. Longer reads and variable insert sizes are likely to greatly facilitate the accuracy and quality of *de novo *assemblies of the mouse genome, which, being homozygous and inbred, should be significantly easier to assemble than those of humans or other experimental vertebrate models.

### Using short read-sequencing data to help rank candidate genes in QTL intervals

Analysis of the phenotypic differences between mouse strains is a powerful way of identifying new disease genes and mechanisms. Here we show that sequencing of a whole mouse chromosome is a powerful approach for variant discovery that facilitates the ranking of candidate genes within QTL intervals (Figure 8). This approach is generally applicable across the many mouse QTLs that have been collected over many decades of mouse genetics.

## Conclusions

We have shown that whole chromosome, and by inference whole genome, sequencing of mouse strains is a viable approach for generating dense maps of genetic variation between mouse strains. We have also shown for the first time that the accuracy of novel SNP discovery from short-read sequencing can be almost as high as the typing of known SNPs, given the proper quality controls are applied. We demonstrate the value of using heterozygous SNP density, when combined with read depth, to predict copy number gains, and suggest that such an approach could be generalized to other inbred genomes. We also illustrate what can be achieved with short read sequencing technologies now, and with the expected increases in read lengths and sequence yield from these technologies complete *de novo *assemblies of mouse strain genomes will soon be well within reach. We show that whole chromosome sequencing can be applied to derive a high-resolution picture of QTL regions and helps to rank candidate genes for follow-up analysis.

## Materials and methods

### Mice

Pedigreed A/J mice were obtained from MRC-Harwell (Oxfordshire, UK), who derived these mice from the Jackson Labs (Bar Harbour, Maine, USA). CAST/Ei mice were also derived from Jackson Lab stock but had been maintained as an independent colony for several years. Mice were genotyped with a set of polymorphic markers to confirm their genetic purity. Murine embryonic fibroblasts were prepared from E14.5 embryos using standard procedures.

### Chromosome isolation

Mitotic cells were arrested by treatment with 0.1 μg/ml demecolcine for 8 hours. Chromosomes were isolated and stained as described previously [17] using a flow cytometer (MoFlo, DAKO, Glostrup, Produktionsvej, Denmark) equipped with two Innova 300 series lasers (Coherent, Santa Clara, CA). Sorted chromosomes were treated with proteinase K and sodium lauroyl sarcosine followed by phenylmethylsulphonyl fluoride before recovering DNA by precipitation.

### Paired-end library preparation and sequencing

Libraries were prepared and sequenced essentially as described [18]. Briefly, 300 ng of isolated chromosomes were fragmented using a disposable nebulizer (Invitrogen, Paisley, Scotland, UK) and purified using a qiaquick column (Qiagen, Valencia, CA, USA). DNA was end-repaired as described and an adaptor ligated to the ends of the DNA (adaptor sequences: 5'ACACTCTTTCCCTACACGACGCTCTTCCGATCxT (x = phosphorothioate bond) and 5'-phosphate-GATCGGAAGAGCGGTTCAGCAGGAATGCCGAG [18]. Fragments of approximately 200 bp were gel-purified and PCR amplified. Flow cells were prepared, clusters generated, and processed Flowcells were sequenced on an Illumina Genome Analyzer 2 and data analyzed using standard methods (Illumina, Little Chesterford, Essex).

### Mapping of sequence reads and analysis of mapping

Reads with uncalled bases were removed from analysis and the remaining reads mapped to the build 37 of the NCBI mouse reference sequence, using MAQ version 0.6.6, with the insert size parameter set to 200 bp and duplicates removed, and using an expected heterozygous rate of 1 in 10,000.

Nominal mean sequence depth was calculated as:

Actual mean sequence depth was calculated as:

Contiguous blocks of sequence were calculated by moving through an ordered list of mapped reads, adding overlapping reads to the current block. When non-overlapping reads were encountered, a new block was initiated.

### SNP calling and confirmation

SNPs were called from mapped sequence data using standard parameters, except that a heterozygous SNP rate of 1/10,000 was used as described above [19]. SNPs were filtered using the MAQ SNP filter using a minimum maximum mapping quality of 60 and maximum depth of 50 (for A/J) or 85 (for CAST/Ei). SNPs were downloaded from dbSNP (version 126). HapMap SNPs were downloaded from [20] and genome coordinates converted to version 37 of the mouse reference genome using the LiftOver tool [33]. In order to select novel SNPs for confirmation, the range of quality scores for SNPs was divided into 6 equally sized bins and 50 SNPs from each bin selected at random. Three-hundred SNPs present in dbSNP but not in the filtered MAQ calls were selected at random. SNPs were genotyped using the Sequenom genotyping platform. P-scores for non-genotyped SNPs were calculated as:

### Indel calling and comparison with capillary data

Indels were called using the samtools package, version 0.1.4 [34], and filtered using the samtools indelFilter script. Indels were called from capillary data downloaded from the Celera website (see below) using ssahaSNP [35]. ssahaSNP output was filtered so that only indels predicted by more than one read were considered. When comparing the two methods, a window of 18 bp in each direction was allowed due to differences in how the two algorithms assign coordinates to the indels.

### Calling of structural variants

An average and standard deviation for the insert size was calculated by fitting a normal distribution to the main peak of a histogram of all insert sizes. Read pairs where the average insert size was greater than the average plus four standard deviations were isolated. Read pairs were allocated to 'clusters' based on whether they overlapped and if the difference in their end points was not more than the mean insert length plus four standard deviations. Overlapping clusters were then overlapped. Only those clusters containing two or more read pairs were retained for further analysis.

We constructed a HMM to detect copy number gains and losses from the alignments of the sequence reads to the reference genome. As previously stated, the underlying assumption is that regions with copy number gains will have an integral increase in sequence depth and regions with copy number losses will have an integral decrease in depth. Additionally, duplicated regions can appear to contain heterozygous SNPs, a fact our model exploits. The input to the HMM is the sequence read depth, the number of heterozygous SNPs and the average number of hits per read calculated over 1-kb windows of the reference sequence. Initially, the parameters of the model are learned using the Baum-Welch and Viterbi training algorithms, respectively. The state classifications are then determined using the Viterbi algorithm. These classifications are post-processed to extract high-confidence calls of at least 10 kb in size.

### Confirmation of deletions identified by aberrantly mapped read-pairs

Deletions predicted in the A/J sequence that overlapped with Ensembl exons (V50) were selected for confirmation. Primers that flanked the deleted region were selected using the Primer3 software. Regions were amplified by PCR using standard protocols from both A/J and C57BL/6J genomic DNA. PCR products were treated with Exonuclease I and Shrimp alkaline phosphatase for 2 hours and then sequenced from both ends with the same primers, using capillary sequencing technologies.

### *De novo *assemblies

For *de novo *assembly of chromosome 17 data we used a new short read assembler: Fuzzypath [26]. For Illumina data, the assembly process consists of two steps: read extension and then assembly. Using the de Bruijn graph algorithm, raw Illumina reads are first extended into segments of consensus sequences each with a maximum length of 2 kb. Sequence extension starts from kmer seeds, which are randomly sampled to ensure overlaps among extended segments. These extended reads are then presented to the Phusion assembler. In the case of A/J and CAST/Ei these extended reads had between 10 and 15× coverage of the reference genome assembly. To generate a hybrid assembly for A/J, 11.6 million mouse whole-genome shotgun reads were aligned to the reference NCBI_M37.fa and only those reads (357,100) that could be uniquely placed on chromosome 17 were combined with the extended reads, described above, and presented to the Phusion assemblier. Velvet [27] and AbySS [28] were used as described.

### Data availability

The sequence data generated in this study have been submitted to the European Short Read Archive (ERA) [ERA000077]. SNPs have been submitted to dbSNP under the handle SC_MOUSE_GENOMES. A/J SNPs have dbSNP accessions starting from [dbSNP:ss130462797] and CAST/Ei SNPs start from [dbSNP:ss130459410].

Assemblies are available at [29]. Additional data files can be downloaded from [36].

## Abbreviations

aCGH: microarray-based comparative genomic hybridization; CNV: copy number variation; HapMap: haplotype map; HMM: hidden Markov model; indel: insertion and deletion mutations; MAQ: Mapping and Assembly with Quality; MHC: major histocompatibility complex; QTL: quantitative trait locus; SNP: single nucleotide polymorphism.

## Authors' contributions

JS, JTS, TK, ARG, MEH, KW, HL, ZN and RD conceived and performed data analysis. DL, LT, and SDB provided mice and generated cell lines for flow sorting. JHN and CMC generated the A/J chromosome substitution strains and phenotyped the mice. IMS and DJA conceived the study, analyzed the data, and wrote the paper. All authors read and approved the final manuscript.

## Additional data files

The following additional data are available with the online version of this paper: a spreadsheet showing the effects of applying different p score thresholds on the accuracy and sensitivity of SNP calling from A/J sequence (Additional data file 1); a spreadsheet showing the effects of applying different p-score thresholds on the accuracy and sensitivity of SNP calling from CAST/Ei sequence (Additional data file 2); a presentation showing analysis of the accuracy of indel calling from A/J Illumina and capillary data (Additional data file 3); a spreadsheet listing A/J deletions identified using read pairs by comparison to the reference C57BL/6J genome (Additional data file 4); a spreadsheet listing CAST/Ei deletions identified using read pairs by comparison to the reference C57BL/6J genome (Additional data file 5); a spreadsheet listing variants predicted by HMM structural variants (both amplifications and deletions) predicted by the HMM based on mapping depth and heterozygotes (Additional data file 6); a spreadsheet listing predicted coding SNP differences in A/J (Additional data file 7); a spreadsheet listing predicted coding SNP differences in CAST/Ei (Additional data file 8); a spreadsheet listing genes affected by predicted structural variants (Additional data file 9).

## Supplementary Material

Additional data file 1 Effects of applying different p score thresholds on the accuracy and sensitivity of SNP calling from A/J sequence. Click here for file

Additional data file 2 Effects of applying different p-score thresholds on the accuracy and sensitivity of SNP calling from CAST/Ei sequence. Click here for file

Additional data file 3 Analysis of the accuracy of indel calling from A/J Illumina and capillary data. Click here for file

Additional data file 4 A complete list of deletions in A/J passing quality control filters, predicted by examining aberrantly mapping read pairs to the C57BL/6J genome. The list includes predicted start and end coordinates, the length of the deletion and the number of read pairs that predict the deletion. Click here for file

Additional data file 5 A complete list of deletions in A/J passing quality control filters, predicted by examining aberrantly mapping read pairs to the C57BL/6J genome. The list includes predicted start and end coordinates, the length of the deletion and the number of read pairs it is predicted by. Click here for file

Additional data file 6 Variants predicted by HMM structural variants (both amplifications and deletions) predicted by the HMM based on mapping depth and heterozygotes. Click here for file

Additional data file 7 Single nucleotide differences between C57BL/6J and A/J that affect Ensembl coding exons, marked as either synonymous, non-synonymous or splice site changes. Also indicated are the sequencing depth and phred-like quality score. Click here for file

Additional data file 8 Single nucleotide differences between C57BL/6J and CAST/Ei that affect Ensembl coding exons, marked as either synonymous, non-synonymous or splice site changes. Also indicated are the sequencing depth and phred-like quality score. Click here for file

Additional data file 9 Genes affected by predicted structural variants (predicted by both read-pair mapping and the HMM) in both strains. The genes, strain, type of variant and exons affected are indicated. Type is either deletion (as predicted from read-pair mapping), copy number loss (CN_Loss) or copy number gain (CB_Gain). Click here for file
