# Supplementary material for: Deep short-read sequencing of chromosome 17 from the mouse strains A/J and CAST/Ei identifies significant germline variation and candidate genes that regulate liver triglyceride levels
Source: Genome Biol. 2009 Oct 13;10(10):R112. doi: 10.1186/gb-2009-10-10-r112 (PMC2784327; doi:10.1186/gb-2009-10-10-r112)
Supplement: Additional data file 3 — Analysis of the accuracy of indel calling from A/J Illumina and capillary data. [file gb-2009-10-10-r112-S3.ppt]

## Slide 1
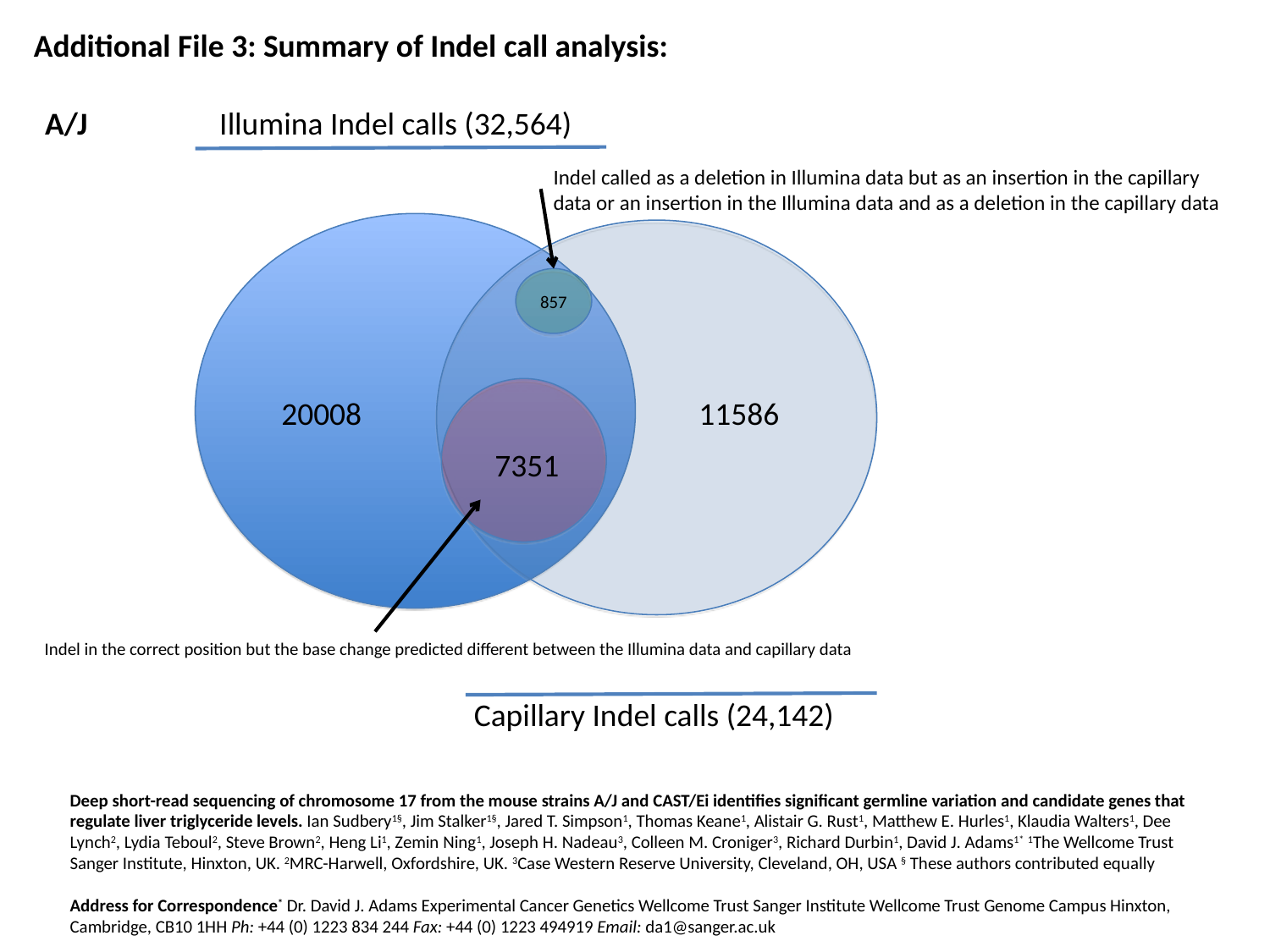

Additional File 3: Summary of Indel call analysis:
Illumina Indel calls (32,564)
A/J
Indel called as a deletion in Illumina data but as an insertion in the capillary data or an insertion in the Illumina data and as a deletion in the capillary data
857
20008
11586
7351
Indel in the correct position but the base change predicted different between the Illumina data and capillary data
Capillary Indel calls (24,142)
Deep short-read sequencing of chromosome 17 from the mouse strains A/J and CAST/Ei identifies significant germline variation and candidate genes that regulate liver triglyceride levels. Ian Sudbery1§, Jim Stalker1§, Jared T. Simpson1, Thomas Keane1, Alistair G. Rust1, Matthew E. Hurles1, Klaudia Walters1, Dee Lynch2, Lydia Teboul2, Steve Brown2, Heng Li1, Zemin Ning1, Joseph H. Nadeau3, Colleen M. Croniger3, Richard Durbin1, David J. Adams1* 1The Wellcome Trust Sanger Institute, Hinxton, UK. 2MRC-Harwell, Oxfordshire, UK. 3Case Western Reserve University, Cleveland, OH, USA § These authors contributed equally
Address for Correspondence* Dr. David J. Adams Experimental Cancer Genetics Wellcome Trust Sanger Institute Wellcome Trust Genome Campus Hinxton, Cambridge, CB10 1HH Ph: +44 (0) 1223 834 244 Fax: +44 (0) 1223 494919 Email: da1@sanger.ac.uk
